# Virtual Reality Mood Induction and Body Image Disturbance in Adolescents With Eating Disorders: An Experimental Study

**DOI:** 10.1002/cpp.70328

**Published:** 2026-09-07

**Authors:** Diana Burychka, Marta Miragall, Maite Zapata, Judith Alvarez‐Borillo, Rosa M. Baños

**Affiliations:** ^1^ Polibienestar Research Institute University of Valencia Valencia Spain; ^2^ Department of Personality, Evaluation and Psychological Treatment, Faculty of Psychology and Speech Therapy University of Valencia Valencia Spain; ^3^ CIBERObn Physiopathology of Obesity and Nutrition Instituto de Salud Carlos III Madrid Spain; ^4^ Acute Child and Adolescent Hospitalization Unit Hospital Clínico Universitario Lozano Blesa Zaragoza Spain

**Keywords:** eating disorders, mood induction, negative affect, positive affect, virtual reality

## Abstract

Eating disorders (EDs) are severe psychiatric conditions characterized by body image disturbance, yet the role of affective states in the maintenance of body image symptoms among adolescents remains poorly understood. Virtual reality–based mood induction procedures (VR‐MIPs) offer a controlled method for examining how experimentally induced affect influences body image outcomes. This within‐subjects experimental study tested the hypotheses that a positive‐MIP would improve, and negative MIP would worsen, body image disturbance indices in female adolescents with EDs. Forty‐two female adolescents diagnosed with an ED, predominantly anorexia nervosa (92.85%), (Age: *M* = 15.88, *SD* = 1.40, BMI: *M* = 18.30, *SD* *=* 2.60) completed two experimental sessions involving positive and negative VR‐MIPs. Assessments included perceived and ideal body size estimation, depressive symptoms, state affect and body satisfaction. The positive‐MIP successfully induced the intended affective changes and produced increases in ideal body size estimation and reductions in body dissatisfaction. Although these effects did not survive conservative correction for multiple comparisons, the converging effect‐size and Bayesian estimates provide preliminary evidence that should be examined in adequately powered samples. In contrast, the negative‐MIP did not produce significant body image‐related changes, and the manipulation check did not confirm the intended mood induction, limiting the interpretability of findings for this condition. These findings point to the potential value of incorporating positive affect induction strategies within body image interventions for female adolescents with EDs. Replication in larger, diagnostically diverse samples and inclusion of male and non‐binary adolescents are needed to consolidate these preliminary findings.

**Trial Registration:**

ClinicalTrials.gov identifier: NCT04617886

## Summary

- Positive affect may play a clinically meaningful factor in body image disturbance among adolescents with eating disorders, and therapeutic approaches that actively foster positive emotional states, not only target negative affect, may help reduce body image disturbance in this population.
- Adolescents with eating disorders may habitually suppress or disengage from negative emotions; clinicians should consider when designing and interpreting emotion‐focused assessments and interventions.
- Virtual Reality may offer a more ecologically valid and potentially more sensitive tool than self‐report measures alone for assessing state‐dependent body image disturbance, and its integration into clinical assessment protocols should be considered.
- Body image disturbance in adolescents with eating disorders may fluctuate with affective state, reinforcing the importance of assessing body image dynamically and in relation to emotional context, rather than as a fixed trait.
- Adaptive emotion regulation, including broadening attention away from disliked body areas, though still preliminary, represents a promising therapeutic mechanism that clinicians may target to reduce body image disturbance in eating disorder treatment.

Eating disorders (EDs) are severe mental conditions characterized by significant physical and psychological impairment (American Psychiatric Association [APA] 2013). Predominantly affecting women and typically emerging during adolescence (Herpertz‐Dahlmann 2009), EDs are associated with high rates of chronicity, relapse and mortality (Fairburn et al. 2015; Franko et al. 2013; Stice et al. 2009), with considerable consequences for quality of life. Additionally, recent epidemiological data indicate that the incidence of EDs in adolescents has risen markedly in the wake of the COVID‐19 pandemic, further underscoring the urgency of identifying maintenance factors in this age group (Devoe et al. 2023).

Body image disturbance is widely recognized as a key risk and maintenance factor in EDs (Sattler et al. 2020), encompassing two interrelated dimensions (Garner and Garfinkel 1981): (1) the affective‐cognitive‐attitudinal component reflecting thoughts, evaluations, and feelings toward one's body, such as body dissatisfaction and shame (Hagman et al. 2015; Sharpe et al. 2018); and (2) the perceptual component referring to inaccuracies in estimating one's body size, weight and shape, such as body overestimation (Cornelissen et al. 2018; Mölbert et al. 2018). Critically, body image disturbance has been shown to persist following treatment (Engel and Keizer 2017; Eshkevari et al. 2014), contributing to relapse (Glashouwer et al. 2019). This underscores the importance of identifying factors that sustain body image disturbance in order to develop more effective interventions (Glashouwer et al. 2019; Jansen et al. 2016).

In addition to body image disturbance, a large body of evidence suggests that disturbances in affect may be central to ED onset and maintenance (Haynos and Fruzzetti 2011; Leppanen et al. 2022; Svaldi et al. 2012). In particular, negative affect (NA)—encompassing distress and negative mood states—has been implicated in both the development and perpetuation of body image disturbance (e.g., Kenny et al. 2022; Engel et al. 2013; Sharpe et al. 2018; Vanzhula et al. 2020).

Experimental evidence suggests that body image is not a static construct but is susceptible to fluctuation in response to internal and external influences (Farrell et al. 2005), including negative affective states (e.g., Baker et al. 1995; Taylor and Cooper 1992), food intake (e.g., Thompson et al. 1993; Vocks et al. 2007) and body image‐related emotional contexts (Gutiérrez‐Maldonado et al. 2010) have been found to modify body image disturbance. Such contextual cues may trigger attentional biases toward body‐related information (Lee and Shafran 2004; Smeets et al. 2008; Smeets et al. 2011) or amplify negative emotional responses, resulting in increased body size overestimation (Smeets et al. 2011; Taylor and Cooper 1992).

Despite the accumulating evidence on NA, the influence of positive affect (PA) on body image disturbance remains considerably less studied. Positive emotions may buffer the effects of NA (Fredrickson 2001) and foster health‐promoting behaviours that support ED recovery (Cardi et al. 2015; Tylka and Wood‐Barcalow 2015). Despite this, experimental research on the role of state positive affect in body image disturbance remains scarce, particularly in clinical adolescent samples, for whom emotion regulation is especially challenging (Ahmed et al. 2015). A more comprehensive understanding of how both PA and NA influence body image could help account for the variability in existing findings and inform the development of more targeted and effective interventions (Peñate et al. 2020; Tiggemann 2010).

The present study therefore aimed to examine the effects of positive and negative mood induction procedures (MIPs) on body image disturbance in adolescents with EDs. VR was employed to enhance immersion and improve the reliability of body size estimation (Diniz Bernardo et al. 2021; Purvis et al. 2015), and a within‐subjects design was adopted to capture intraindividual changes across the two MIP conditions. Based on the preceding literature, two hypotheses were formulated: (H1) a positive‐MIP will improve both the affective and perceptual dimensions of body image disturbance; and (H2) a negative‐MIP will worsen both dimensions of body image disturbance.

## 1 Materials and Measures

### 1.1 Participants

The sample comprised 42 female adolescents (*M* = 15.88; SD = 1.40) recruited from three clinical settings in Spain: the Eating Disorders Unit of the Psychiatry Department of the Hospital Clínico Universitario Lozano Blesa (*n* = 40), the Mental Health Ward of the Hospital Universitario Doctor Peset (*n* = 1) and at Ita PREVI Clinical Psychology Center (*n* = 1), all located in Spain.

Inclusion criteria were (1) age between 13 and 18 years; (2) a confirmed diagnosis of anorexia nervosa, bulimia nervosa or unspecified feeding or eating disorder per DSM‐5 (APA 2013) and/or ICD‐10 (WHO 1992) diagnostic criteria; and (3) a Beck Depression Inventory‐II (BDI‐II; Beck et al. 1996) score not exceeding 28, verified by the participant's attending clinician.

Exclusion criteria comprised: (1) elevated suicide risk, as indicated by a BDI‐II score above 28 (Beck et al. 1996) and confirmed by a mental health professional; (2) Body Mass Index (BMI) below 14 or above 28; (3) comorbid severe mental disorder with psychotic or manic features, epilepsy or sensory impairments; (4) substance abuse; and (5) pregnancy.

Sample size was estimated using G*Power (v.3.1; Faul et al. 2007), requiring 42 participants to detect a moderate effect size (*d* = 0.40) with 95% power at *α* = 0.05, based on prior comparable studies (e.g., Baker et al. 1995; Plies and Florin 1992; Taylor and Cooper 1992).

All participants provided written informed consent in accordance with the Declaration of Helsinki. The study was approved by the Ethics Committees of the University of Valencia (No. 1127840) and Hospital Universitario Doctor Peset (No. 23/21). This study is registered with ClinicalTrials.gov, number NCT04617886.

### 1.2 Measures

#### 1.2.1 Sociodemographic and Anthropometric Data

Basic participant information was collected via a brief self‐administered form, including age, educational level, occupation, presence of psychiatric and/or physical conditions and self‐reported weight and height.

#### 1.2.2 Self‐Reports

The Beck Depression Inventory‐II (BDI‐II; Beck et al. 1996; Sanz et al. 2003) a 21‐item self‐report instrument measuring the severity of depressive symptoms over the preceding 4 weeks, with total scores ranging from 0 to 63. The Spanish adaptation showed high internal consistency in psychiatric samples (*α* = 0.92). In the present study, Cronbach's *α* was 0.77 and 0.78 prior to the positive‐ and negative‐MIP conditions, respectively.

Positive and negative affect. The positive and negative affect scales (PANAS; Watson et al. 1988; López‐Gómez et al. 2015). The PANAS consists of two scales: positive affect scale (PA) and negative affect scale (NA), each consisting of 10 items. The items are scored using a 5‐point Likert scale (1, *not at all or very slightly* to 5, *very much*) in the last month. The two scores are obtained with the sum of the scores of each of the items on the scale. In the present study, the scale showed an adequate internal consistency (*α* = 0.91 for PA and 0.86 for NA).

The Body Image States Scale (BISS; Cash 2002; translated by the study authors) is a six‐item self‐report measure assessing state body satisfaction on a 9‐point Likert scale. The BISS was administered immediately before and after each MIP session. Internal consistency was acceptable across administrations, with Cronbach's *α* ranging from 0.76 to 0.88 depending on the condition and time point (positive‐MIP: *α* = 0.85 pre, *α* = 0.88 post; negative‐MIP: *α* = 0.76 pre, *α* = 0.85 post).

#### 1.2.3 VR Tasks

##### 1.2.3.1 Body Size Estimation of the Perceived and Ideal Body

**FIGURE 1:**
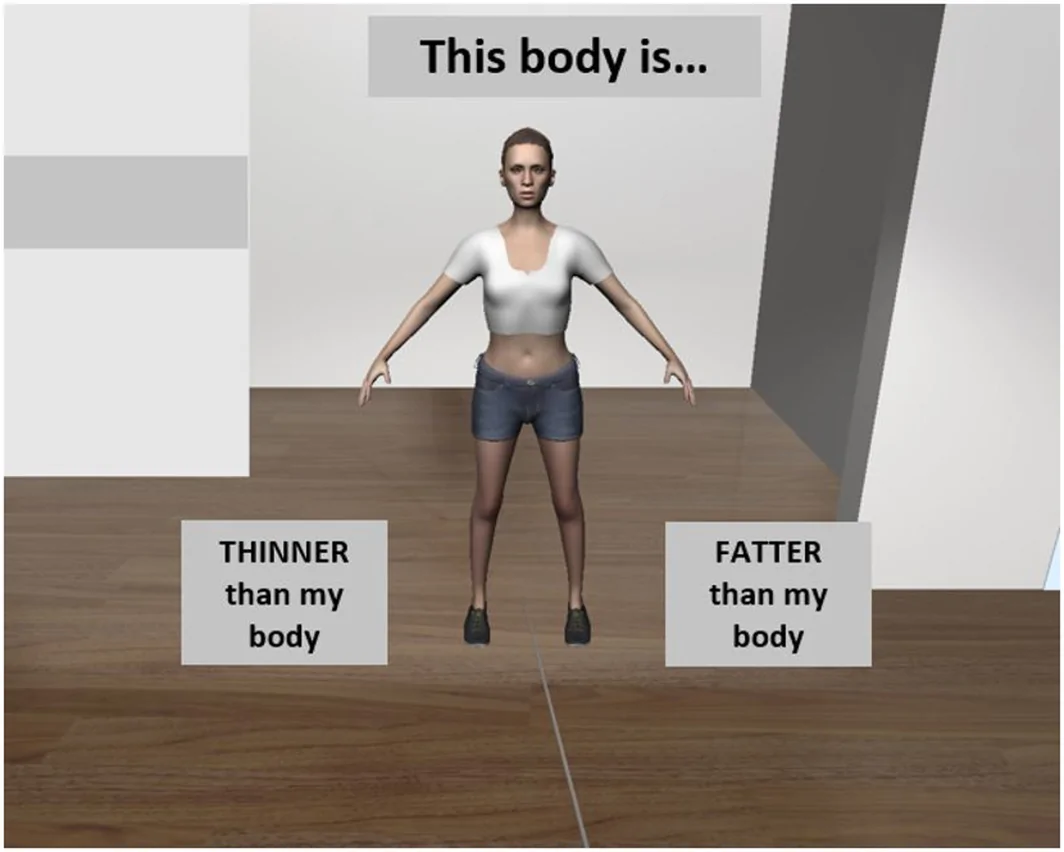
Virtual environment displayed during the task of perceived body size estimation.

This task was adapted from Gledhill et al. (2017). Participants were presented with a series of avatars and asked to judge whether each appeared ‘fat’ or ‘thin’ relative to their perceived or ideal body (see Figure 1). The task consisted of two sequential phases: (1) eight avatars spanning BMI values from 14 to 29 kg/m^2^, each displayed 10 times in randomized order; and (2) five avatars with BMI values within ±2 units of the cut‐off identified in Phase 1. The perceived and ideal body size estimation tasks were administered in counterbalanced order, with participants completing both tasks on four occasions: before and after the positive‐MIP and before and after the negative‐MIP. The primary outcome was an estimated BMI for both perceived and ideal body size for each participant.

##### 1.2.3.2 VR‐Based Body Dissatisfaction

Body dissatisfaction was operationalized as the discrepancy between perceived and ideal body size estimations derived from the VR tasks, calculated as follows: *Perceived Body Size Estimation—Ideal Body Size Estimation = Body Dissatisfaction*. Higher scores indicate greater body dissatisfaction (e.g., BMI 20—BMI 18 = 2).

##### 1.2.3.3 Positive and Negative‐MIPs Through VR

Mood induction was delivered via two VR scenarios set in a virtual urban park. During each MIP, participants walked through the park and completed tasks designed to elicit either a positive (i.e., happiness) or negative (i.e., sadness) affective state: (1) a Velten‐style self‐referential mood induction task (Velten 1968); (2) viewing affectively valenced images selected from the International Affective Picture System (IAPS; Lang et al. 2005); (3) listening to a musical excerpt and viewing a brief film scene chosen for their mood‐eliciting properties (Donen et al. 1952; Eich and Metcalfe 1989; Gross and Levenson 1995); and (4) recalling a personally relevant autobiographical memory.

### 1.3 Virtual Reality Environment Configuration

A set of 19 computer‐generated female bodies was developed using MakeHuman (MakeHuman 1.1.1 r2128; The MakeHuman team) and Autodesk 3ds Max (Autodesk 3ds Max 2017 19.0 SP3; Autodesk, San Rafael, CA). Avatar BMI values ranged from 14 kg/m^2^ (severe underweight) to 29 kg/m^2^ (overweight) consistent with WHO (2004) classification criteria. Physical attributes including height, weight, muscle mass and body proportions were adjusted to maximize realism. To ensure visibility of body areas most relevant to appearance evaluation (e.g., abdomen and thighs), avatars were dressed in a white top and blue shorts.

The VR environment was built in Unity3D (v5.6.0f3; Unity Technologies, San Francisco, CA) and displayed through Oculus Rift and Oculus Rift S head‐mounted displays (Oculus VR, Irvine, CA), providing a resolution of 2160 × 1200, a 110° field of view, and a 90‐Hz refresh rate. The system was run on a high‐performance workstation equipped with a quad‐core Intel Core i5‐7640X processor (4.00 GHz), 16 GB RAM and an NVIDIA GeForce GTX 1070Ti graphics card with 8 GB GDDR5 memory.

### 1.4 Procedure

The study was structured into two phases: (1) screening and (2) two experimental sessions of approximately 90 min each. Participants who met the inclusion criteria provided informed consent and completed a baseline battery of questionnaires administered online via LimeSurvey. For ethical reasons, the BDI‐II was readministered prior to each experimental session to monitor depressive symptomatology. Sessions were cancelled or rescheduled if a participant's score exceeded 28, indicating severe depression symptomatology.

During each session, participants first completed the pre‐MIP measures (BISS and VR‐body size estimation tasks for both perceived and ideal body). They were then randomly assigned to either the positive‐ or negative‐MIP condition using Random Allocation Software (v.2.0; Saghaei 2004). MIPs were administered in the absence of the researcher to minimize demand characteristics. Upon completion of the MIP, participants repeated the post‐induction measures (BISS and VR‐body size estimation). The two sessions were scheduled approximately 1 week apart, with a minimum interval of 5 days, and each participant received the alternate MIP condition in the second session.

To address ethical concerns regarding potential mood deterioration, a mood repair procedure was incorporated at the end of the negative‐MIP session, consisting of exposure to the positive‐MIP. Upon completion of both sessions, all participants received a thorough debriefing to address any residual effects of the experimental manipulations.

### 1.5 Data Analyses

All statistical analyses were performed using SPSS (v.28; IBM Corp.), and Bayesian analyses were conducted in JASP (v.0.16.4; JASP Team 2022). Descriptive statistics were computed to characterize the sample across all study variables. Univariate normality was evaluated through skewness and kurtosis indices, revealing no severe departures from normality (Kline 2005). The associations between study variables were examined using Pearson correlation coefficients.

The effectiveness of the MIPs was assessed by comparing PA and NA pre‐ to post‐induction using paired‐sample *t*‐tests. The same approach was employed to examine the effect of each MIP condition on the dependent variables. To control the Type I error rate arising from multiple paired comparisons, the Holm–Bonferroni correction (Holm 1979) was applied within three prespecified families of tests: (1) the four manipulation‐check tests (PA and NA, positive and negative‐MIP; Table 2), (2) the four outcome tests conducted within the positive‐MIP condition and (3) the four outcome tests conducted within the negative‐MIP condition (Table 3).

Given the conservative nature of family‐wise error corrections such as Holm–Bonferroni, and considering our sample size, we additionally report a supplementary analysis (Benjamini and Hochberg 1995) to examine whether potentially meaningful effects might be masked by reduced statistical power. Finally, because frequentist inference only provides evidence against the null hypothesis and cannot provide probabilistic evidence in favour of it, we complemented the analyses with Bayesian paired‐sample *t*‐tests for all comparisons, following recommended practice (Hoijtink et al. 2019; Keysers et al. 2020). Analyses used the default Cauchy prior (*r* = 0.707; Rouder et al. 2009), and we report BF₁₀, which quantifies the relative evidence for the alternative hypothesis (a pre–post change) over the null hypothesis (no change). A Bayes factor of 1 is considered no evidence, between 1 and 3 anecdotal, between 3 and 10 moderate, between 10 and 30 strong, between 30 and 100 very strong and more than 100 extreme evidence that the data are more likely under the alternative hypothesis (Wagenmakers et al. 2018). Effect sizes were calculated using Cohen's *d*, interpreted according to conventional benchmarks: small (*d* = 0.20), moderate (*d* = 0.50) and large (*d* = 0.80; Cohen 1988). The significance threshold was set at *p* < 0.05, Holm‐corrected within family, for all frequentist analyses. Missing data were minimal and are reported descriptively in Table 1. No formal imputation procedure was applied given the limited extent of missingness.

**TABLE 1:** Baseline sociodemographic, clinical characteristics of participants (*n* = 42).

Baseline characteristic	*M* (*SD*)/%
Age^a^	15.88 (1.40)
BMI	18.30 (2.60)
**Sociodemographic**	
Highest educational level	
Primary school	42.90
Middle school	57.10
Employment status	
Student	97.60
Employed	2.40
**Clinical**	
Previous psychological treatment^b^	35.70
Medication treatment^c^	76.19
ED diagnosis	
AN	92.85
BN	2.38
USFED	4.76
Years since the diagnosis	
< 6 months	9.50
Between 6 months and 1 year	4.80
Between 1 and 2 years	54.80
Between 2 and 3 years	19.10
> 3 years	7.10

*Note:* ^a^ *n* = 41 (one missing value due to nonresponse). ^b^ *n* = 36 (six missing values due to nonresponse). ^c^ Reflects the percentage of participants who have prescribed medication as part of their mental health treatment.

Abbreviations: AN = anorexia nervosa; BMI = body mass index; BN = bulimia nervosa; ED = eating disorders; USFED = unspecified feeding and eating disorders.

## 2 Results

### 2.1 Descriptive Statistics

Means and standard deviations for all sociodemographic, anthropometric and clinical variables are presented in Table 1.

### 2.2 Manipulation Check Analyses: Testing the Efficacy of the Positive and Negative‐MIP in Changing PA and NA

The positive‐MIP significantly increased PA and decreased NA (both Holm‐corrected *p*s < 0.001, *d*s = −0.79 and 1.06, respectively), a result unchanged under the FDR robustness check and supported by extreme Bayesian evidence (BF₁₀ = 2009.66 and 3.89 × 10^5^, respectively). In contrast, the negative‐MIP produced no significant change in PA (*p* = 0.941; BF₁₀ = 0.17, moderate evidence favouring the null) and only a nonsignificant trend for NA (*p* = 0.055; Holm‐corrected *p* = 0.110; BF₁₀ = 0.99, inconclusive), indicating that the intended affective state was not induced (see Table 2). Because the manipulation check failed for the negative‐MIP condition, the outcome comparisons within that condition (below) were corrected for completeness but were not interpreted as a formal test of H2.

**TABLE 2:** Differences in pre‐postinduction PA and NA per condition.

	Pre‐MIP		Post‐MIP		*t*	*df*	*p*	*p* (Holm)	Cohen's *d*	95% CI		BF_10_
	*M*	*SD*	*M*	*SD*						*LL*	*UL*	
**Positive‐MIP**												
PA	26.61	9.02	30.00	9.28	−5.06	40	< 0.001	< 0.001	−0.79	−1.14	−0.43	2009.66 (1.85 × 10 ^−7^)
NA	24.02	8.32	17.31	6.55	6.80	40	< 0.001	< 0.001	1.06	0.67	1.44	3.89 × 10^5^ (3.51 × 10 ^−11^)
**Negative‐MIP**												
PA	26.47	8.31	26.40	9.36	0.08	39	0.941	0.941	0.01	−0.30	0.32	0.17 (0.05)
NA	24.62	8.25	22.40	7.62	1.98	39	0.055	0.110	0.31	−0.07	0.63	0.99 (0.02)

*Note: p* values are Holm–Bonferroni corrected within this family. FDR‐corrected values (Benjamini–Hochberg) were < 0.001, < 0.001, 0.941 and 0.073, respectively and did not alter any conclusion. BF₁₀ = Bayes factor for a pre–post change relative to the null hypothesis; values in parentheses indicate the proportional numerical error of the estimate.

Abbreviations: PA = positive affect (PANAS); NA = negative affect (PANAS).

### 2.3 Effect of Positive‐ and Negative‐MIP on the Self‐Reported and VR Measures of the Affective and Perceptual Body Image Disturbance

Following the positive‐MIP, uncorrected paired *t*‐tests indicated an increase in ideal body size estimation (*p* = 0.023, *d* = −0.369) and a reduction in VR‐measured body dissatisfaction (*p* = 0.018, *d* = 0.386), with perceived body size estimation and BISS‐state body dissatisfaction not reaching significance (*p*s = 0.172 and 0.084, respectively). After Holm–Bonferroni correction, neither ideal body size estimation nor VR‐body dissatisfaction remained significant (both corrected *p*s = 0.072). However, under this more liberal criterion (i.e., supplementary FDR‐corrected analysis), both effects reached significance (corrected *p*s = 0.046), suggesting they may warrant further investigation into larger samples. Furthermore, Bayesian analyses provided anecdotal evidence for these same two comparisons (ideal body size estimation: BF₁₀ = 2.00; VR‐body dissatisfaction: BF₁₀ = 2.49). Taken together, the positive‐MIP effects on ideal body size estimation and VR‐measured body dissatisfaction should be regarded as preliminary and warrant replication in an adequately powered sample.

Because the negative‐MIP manipulation check did not confirm the intended affective change, the corresponding outcome comparisons cannot be interpreted as a valid test of H2. Descriptively, no pre‐to‐post changes approached significance following the negative‐MIP across any of the outcome measures, perceived body size estimation, ideal body size estimation, VR‐body dissatisfaction or BISS‐state body dissatisfaction (all *p*s > 0.05; Holm‐corrected *p*s ≥ 0.85; see Table 3). Consistent with the absence of a successful mood induction, Bayesian analyses favoured the null hypothesis for these outcomes (BF₁₀ = 0.17–0.35), indicating anecdotal‐to‐moderate evidence that the negative‐MIP produced no effect on body image disturbance.

**TABLE 3:** Results of the paired samples *t*‐tests for perceived and ideal body size estimation and body dissatisfaction (measured by VR and self‐report).

	Pre‐MIP		Post‐MIP		*t*	*df*	*p*	*p* (Holm)	Cohen's *d*	95% CI		BF_10_	
	*M*	SD	*M*	SD						*LL*	*UL*		
**Positive‐MIP**													
Perceived body size estimation	19.79	3.71	19.53	3.71	1.39	41	0.172	0.172	0.215	−0.09	0.51	0.41 (0.04)	
Ideal body size estimation	16.90	1.71	17.25	1.82	−2.36	40	0.023	0.072	−0.369	−0.68	−0.05	2.00 (0.02)	
BISS‐state body dissatisfaction	3.77	1.51	3.96	1.58	−1.77	40	0.084	0.168	−0.277	−0.59	0.04	0.71 (0.03)	
VR‐Body dissatisfaction	2.87	4.03	2.22	4.04	2.47	40	0.018	0.072	0.386	0.07	0.70	2.49 (1.02 × 10 ^−8^)	
**Negative‐MIP**													
Perceived body size estimation	19.75	3.85	19.86	4.78	−0.29	41	0.777	1.00	−0.044	−0.35	0.26	0.17 (0.06)	
Ideal body size estimation	16.95	1.92	17.16	1.88	−1.27	41	0.213	0.85	−0.195	−0.50	0.11	0.35 (0.04)	
BISS‐state body dissatisfaction	3.90	1.42	3.93	1.42	−0.36	40	0.721	1.00	−0.056	−0.36	0.25	0.18 (0.05)	
VR‐body dissatisfaction	2.81	4.11	2.70	5.05	0.22	41	0.828	1.00	0.034	−0.27	0.34	0.17 (0.06)	

*Note:* BF₁₀ = Bayes factor for a pre–post change relative to the null hypothesis; values in parentheses indicate the proportional numerical error of the estimate. For the positive‐MIP family, FDR‐corrected values (Benjamini–Hochberg) were 0.046, 0.046, 0.112 and 0.172 (VR, ideal, BISS and perceived); for the negative‐MIP family, all FDR‐corrected values were 0.828. Negative‐MIP outcome tests are reported for completeness but were not interpreted as a formal test of H2, because the manipulation check did not confirm the intended affective change in this condition (see Table 2); all remained nonsignificant under uncorrected, Holm–Bonferroni and FDR thresholds.

Abbreviations: BISS‐state body dissatisfaction = body image states scale; CI = confidence interval; LL = lower limit; *p* = uncorrected; *p* (Holm) = Holm–Bonferroni corrected within family; UL = upper limit; VR = virtual reality.

## 3 Discussion

The present study examined the relationship between affect and body image disturbance in adolescents with EDs. The first hypothesis received partial, preliminary support: the positive‐MIP robustly increased state PA and decreased state NA; it also showed small‐to‐moderate changes in the expected direction, including an increase in ideal body size estimation and a reduction in VR‐measured body dissatisfaction. However, these effects on body image did not survive correction for multiple comparisons, and Bayesian analyses indicated only anecdotal evidential strength. These findings should therefore be interpreted as preliminary rather than confirmed. Notably, no changes were observed in perceived body size estimation or self‐reported body dissatisfaction.

These findings are consistent with evidence suggesting a protective role of PA on ideal body size perception and body dissatisfaction (Góngora 2014) and contrast with the well‐documented vulnerability‐enhancing effect of NA on the thin ideal (Tylka and Subich 2004). Our results are in line with findings that adaptive emotion regulation preserves positive affect and prevents acute body dissatisfaction (Prefit et al. 2020), suggesting that PA may function as a protective mechanism, whether maintained through regulation strategies or, as in the present study, directly enhanced through mood induction.

From a positive psychology perspective, the ‘undoing hypothesis’ proposes that fostering PA may attenuate risk factors associated with psychopathology (Fredrickson and Levenson 1998; Silk et al. 2006), with potential implications for both prevention and intervention in EDs (Wong et al. 2021). Consistent with this, higher PA may promote broader, less detail‐focused cognitive processing (Hess et al. 2012), which could in turn reduce body image disturbance. This interpretation was informally corroborated during debriefing, with participants reporting a reduced tendency to focus on disliked body areas during the VR tasks, consistent with the broadening effect of PA described by Fredrickson (2001). Taken together, these results suggest that positive mood induction may buffer negative affect and support healthier emotional regulation strategies (Cardi et al. 2015), though the underlying mechanisms warrant further investigation.

The second hypothesis was not supported. Contrary to prior evidence linking experimentally induced NA to increased perceived body size estimation (Fox et al. 2012; Taylor and Cooper 1992) or body dissatisfaction (Haedt‐Matt et al. 2012), the negative‐MIP did not significantly affect either the perceptual or affective dimensions of body image disturbance. However, these results should be interpreted with caution, as the manipulation check did not confirm the expected affective changes following the negative‐MIP. One possible explanation is the use of maladaptive emotion regulation strategies commonly observed in ED populations: Meta‐analyses show that ED population relies heavily on suppression and avoidance of emotions among other maladaptive strategies (Oldershaw et al. 2015; Prefit et al. 2019) as negative mood states are poorly tolerated (Fairburn 2008). This pattern aligns closely with the cognitive‐interpersonal maintenance model of anorexia nervosa (Schmidt and Treasure 2006; Treasure and Schmidt 2013), which identifies harm avoidance as a core premorbid trait that promotes hypervigilance to emotional threat and increasingly rigid avoidance‐based coping (e.g., avoidance of negative affect). This is further supported empirically by Wildes et al. (2010), who demonstrated that emotion avoidance mediates the relationship between negative affect and ED psychopathology specifically in anorexia nervosa, the predominant diagnosis in the present sample (92.85%). Qualitative evidence supports this explanation: Individuals with anorexia nervosa report deliberately inhibiting and masking sadness in front of others (Bryant et al. 2022). Taken together, these findings suggest that individuals with EDs may disengage from or suppress sad mood rather than fully experience it, which could make conventional negative‐MIPs less effective in this population. In light of the transdiagnostic role of NA in EDs, further research examining the impact of negative mood induction in this population is needed.

These findings carry several clinical implications. First, they underscore the importance of targeting affect, particularly PA, within ED interventions for adolescents, highlighting adaptive emotional regulation as a promising therapeutic focus for adolescent populations. In this regard, the participants' tendency to disengage from negative affect is consistent with an increasing evidence that identifies experiential avoidance as a transdiagnostic maintaining mechanism in EDs. This supports the applicability of acceptance‐ and mindfulness‐based approaches that encourage engagement with internal emotional experience (Gulacan et al. 2025). Second, the study contributes to a growing body of evidence supporting VR as a valid assessment tool for both affective and perceptual dimensions of body image disturbance. By providing an immersive, interactive environment that facilitates realistic body representation and embodiment (Cornelissen et al. 2015; Fisher et al. 2020; Wolf et al. 2021), VR addresses key limitations of traditional assessment methods, such as limited reliability and ecological validity (Ferrer‐García and Gutiérrez‐Maldonado 2012). Overall, these findings add support to the notion of fluctuating body representations in EDs (Cash 2002) and highlight the potentially clinically relevant role of positive mood induction in body image disturbance.

Several limitations should be noted in this study. The reliance on self‐report measures introduces the possibility of social desirability bias. Furthermore, only two basic emotional states were examined (i.e., happiness and sadness), and future studies should incorporate other affectively salient states in ED populations, such as anger and shame (Elliott et al. 2020). The sample was also heavily weighted toward a restrictive‐symptom presentation of EDs (92.85% anorexia nervosa), which substantially limits the generalizability of the present findings to the broader ED spectrum, particularly to conditions involving binge‐purge symptomatology. Thus, although broad emotion dysregulation appears largely transdiagnostic across EDs (Prefit et al. 2019), specific affect‐regulation profiles may nonetheless differ (e.g., heightened impulsivity and affect‐driven eating in bulimia nervosa, versus restrictive, avoidant coping in anorexia nervosa; Lavender et al. 2015). The present results should therefore be interpreted as specific to a restrictive‐spectrum ED presentation, and future studies should prioritize more diagnostically balanced recruitment to determine whether the observed asymmetry between positive and negative mood induction generalizes across the ED spectrum. A further limitation concerns the study's statistical power for detecting effects after correction for multiple comparisons: applying the Holm–Bonferroni correction across the four positive‐MIP outcomes meaningfully reduces the power to detect a true effect of the observed magnitude (*d* = 0.37–0.39). This is a common limitations associated with a preliminary research with hard‐to‐recruit clinical adolescent populations, and it does not undermine the value of the effect size and confidence interval estimates reported here; these can directly inform the design of an adequately powered confirmatory trial (estimated *n* around 100–125) to determine whether these preliminary findings replicate.

Future research should continue investigating the relationship between body image disturbance and context‐dependent affective factors, using longitudinal and within‐person network analyses to capture dynamic changes over time. In particular, examining networks of emotional distress, including shame, anxiety and PA, in relation to ED symptoms represents a promising avenue (Elliott et al. 2020; Solmi et al. 2018) including adolescent samples (Kenny et al. 2022). Recent evidence points to guilt and shame as particularly promising intervention targets (Wong et al. 2021). Future studies should also prioritize more balanced diagnostic recruitment, inclusion of male and non‐binary adolescents and the systematic piloting of mood induction protocols in clinical populations prior to their use in experimental designs. Broader sample diversity in terms of age, gender identity and ED diagnosis will be essential to improving the generalizability of findings in this area.

In conclusion, although replication is needed, the present findings suggest that PA may play a meaningful role in body image disturbance in adolescents with EDs, a dimension that has received comparatively less attention in the literature during the last decades. A deeper understanding of the affective processes underlying perceptual and attitudinal body image alterations may contribute to the development of more effective, targeted ED interventions.

## Funding

This work was supported by the MCIN/AEI/10.13039/501100011033 and by FSE Investing in your future under grant PRE2018‐084882.

## Ethics Statement

This study was approved by the Ethics Committees of the University of Valencia (No. 1127840) and Hospital Universitario Doctor Peset (No. 23/21) and was conducted in accordance with the Declaration of Helsinki.

## Consent

Written informed consent was obtained from all participants prior to their inclusion in the study.

## Conflicts of Interest

The authors declare no conflicts of interest.

## Data Availability

The data that support the findings of this study are at doi.org/10.17605/osf.io/4wext.
